# *Citrus junos* Tanaka Peel Extract Exerts Antidiabetic Effects via AMPK and PPAR-*γ* both *In Vitro* and *In Vivo* in Mice Fed a High-Fat Diet

**DOI:** 10.1155/2013/921012

**Published:** 2013-05-22

**Authors:** Sung Hee Kim, Haeng Jeon Hur, Hye Jeong Yang, Hyun Jin Kim, Min Jung Kim, Jae Ho Park, Mi Jeong Sung, Myung Sunny Kim, Dae Young Kwon, Jin-Taek Hwang

**Affiliations:** ^1^Korea Food Research Institute, 1201 Anyangpangyoro, Bundang-gu, Seongnam-si, Gyeonggi-do 463-746, Republic of Korea; ^2^Department of Food Science & Technology, Gyeongsang National University, 501 Jinjudaero, Jinju, Gyeongnam 660-701, Republic of Korea

## Abstract

The antidiabetic effect of the *Citrus junos* Tanaka (also known as yuja or yuzu) was examined. Ethanol extract of yuja peel (YPEE) significantly stimulated 2-[*N*-(7-nitrobenz-2-oxa-1,3-diazol-4-yl)amino]-2-deoxy-D-glucose (2-NBDG) uptake in C2C12 myotubes. However, ethanol extract of yuja pulp (YpEE) and water extract of yuja peel (YPWE) or pulp (YpWE) did not stimulate glucose uptake. In addition, peroxisome proliferator-activated receptor gamma (PPAR-*γ*) and AMP-activated protein kinase (AMPK) activities were increased by YPEE in a dose-dependent manner. Pretreatment of AMPK inhibitor decreased the glucose uptake stimulated by YPEE in C2C12 myotubes. We confirmed the anti-diabetic effect of YPEE in mice fed a high fat-diet (HFD). Compared with control mice on a normal diet (ND), these mice showed increased body weight, liver fat, insulin resistance, triacylglycerol (TG), and total cholesterol content. Addition of 5% YPEE significantly reduced the weight gain and rise in liver fat content, serum triacylglycerol (TG), total cholesterol, and insulin resistance found in mice fed a high-fat diet (HFD). Moreover, YPEE reduced the secretion of HFD-induced adipocytokines such as leptin and resistin. YPEE also resulted in increased phosphorylation of AMPK in muscle tissues. These results suggest that ethanol extract of yuja peel exerts anti-diabetic effects via AMPK and PPAR-*γ* in both cell culture and mouse models.

## 1. Introduction 

Metabolic syndrome is a serious public health problem, characterized by several metabolic risks such as diabetes mellitus, obesity, and hypertension. Diabetes mellitus is characterized by elevated blood glucose levels and is associated with the development of secondary diseases. Available therapeutic agents, including sulphonylureas and thiazolidinediones, have been widely used for treating diabetes; however, most of these drugs are associated with side effects [1]. Therefore, recently, natural products with antidiabetic activity have been highlighted for the treatment of diabetes and are believed to have minimal side effects. These natural products appear to be able to control metabolic syndrome and diabetes by reducing disease-related biomarkers [2, 3].

AMP-activated protein kinase (AMPK) and peroxisome proliferator-activated receptor gamma (PPAR-*γ*) are well-known biomarkers for antidiabetic drugs that contribute to insulin-sensitizing activities [4–6]. AMPK is a metabolic protein that triggers catabolic pathways, including glycolysis, for generating adenosine 5′-triphosphate (ATP). In contrast, anabolic pathways, including fatty acid and cholesterol synthesis, are blocked by AMPK, inhibiting ATP-consumption [4, 5]. Currently, AMPK is believed to act as a marker in the treatment of diabetes using natural ingredients or compounds [4, 5]. Another target for antidiabetic action is PPAR-*γ*, which regulates glucose metabolism via gene expression [6]. Currently, several insulin-sensitizing drugs, including thiazolidinediones, are used to treat diabetes by targeting PPAR-*γ* [7]. As in case of AMPK, a number of ingredients or compounds derived from plants have been developed to treat diabetes by targeting PPAR-*γ* [8, 9].

Development of insulin resistance is also affected by several cytokines, including leptin and resistin [10, 11]. Leptin is believed to play a role in insulin resistance, since its levels are abnormally high in the plasma of diabetic patients [10]. Resistin is an inflammatory cytokine that plays a role in the regulation of glucose metabolism [11] and several studies have suggested that resistin leads to insulin resistance *in vivo* [11, 12].

Yuja (*Citrus junos* Tanaka), also known as yuzu in Japanese, is a yellow-colored citrus fruit that has traditionally been used to improve blood circulation and treat the common cold in Korea, Japan, and China. Recently, yuja has been found to be effective in preventing certain diseases, including those associated with oxidative stress and inflammation [13, 14]. However, its antidiabetic effects have not yet been elucidated.

In this study, we examined the effect of yuja extracts on glucose uptake in C2C12 myotubes via targeting the AMPK and PPAR-*γ* signaling pathways. Furthermore, we demonstrated that yuja extracts improve insulin resistance and reduce adipocytokine production in the mice fed a high-fat diet. 

## 2. Materials and Methods

### 2.1. Materials

C2C12 cells were purchased from the American Type Culture Collection (Manassas, VA, USA). Dulbecco's modified Eagle's medium (DMEM) and fetal bovine serum were purchased from WelGene (Daegu, Republic of Korea). The compound 2-[*N*-(7-nitrobenz-2-oxa-1,3-diazol-4-yl)amino]-2-deoxy-d-glucose (2-NBDG) was purchased from Invitrogen (Carlsbad, CA, USA). Phosphospecific antibodies against AMP-activated protein kinase (AMPK) and beta-actin were purchased from Cell Signaling Technology (Beverly, MA, USA). SuperFect Transfection Reagent was purchased from Qiagen (Valencia, CA). DNA expression plasmids for PPAR-*γ* and RXR-*α* and luciferase reporter vectors containing PPAR-response elements (PPRE) were a kind gift from Dr. Kim Jae Bum (Seoul National University, Republic of Korea). Yuja was provided from Goheung County Office (Republic of Korea), where a voucher specimen was deposited.

### 2.2. Extraction and Lyophilization

Yuja pulp and peel powders (6 kg, resp.) were extracted with 10-fold volume of 70% ethanol or 100% water by shaking for 24 h at 25°C, and precipitates were removed by centrifugation at 8,000 × g for 30 min (Beckman, USA). Supernatants were lyophilized using a freeze dryer (Il Shin, Republic of Korea). 

### 2.3. HPLC Analysis of Flavonoids

A YMC ODS-AM (250 mm × 4.6 mm ID; particle size, 5 *μ*m) reversed-phase column (Kyoto, Japan) was used for HPLC analysis. The mobile phase consisted of solvent A, 0.1% acetic acid in water, and solvent B, 0.1% acetic acid in acetonitrile. The following gradient was used: initial 0 min A/B (88 : 12, v/v), 18 min A/B (78 : 22), 28 min A/B (72 : 28), 35 min A/B (62 : 38), 48 min A/B (52 : 48), 54 min A/B (32 : 68), 58 min A/B (0 : 100), 60 min A/B (0 : 100), and 62 min A/B (88 : 12). The column was equilibrated for 15 min prior to each analysis. The mobile phase flow rate was 1.0 mL/min, the column temperature was 35°C, the injection volume was 20 *μ*L, and the UV detector was operated at 285 nm.

### 2.4. Quantitative Analysis of Phenolic Compounds

Analysis of phenolic compounds was performed using Folin-Ciocalteu reagent. Dry samples (10 mg) were mixed with 1 mL of 70% ethanol, followed by addition of 0.1 mL Folin-Ciocalteu reagent. After 3 min, 10% (w/v) Na_2_CO_3_ was added to each reaction mixture. Reactions were performed in the dark for 60 min, and absorbance at 725 nm was recorded using a UV spectrophotometer (JASCO, Japan). Total phenolic compound contents were quantified according to a calibration standard curve of caffeic acid (2–10 mg/L). 

### 2.5. Muscle Differentiation and Glucose Uptake Assay

C2C12 cells were maintained in DMEM containing 10% fetal bovine serum in a CO_2_ incubator. Differentiation was induced by incubation with normal medium containing 1% horse serum for 6 days. Fully differentiated cells were incubated overnight in serum-free medium containing low glucose and were added to 2-[*N*-(7-nitrobenz-2-oxa-1,3-diazol-4-yl)amino]-2-deoxy-D-glucose (2-NBDG) in the presence or absence of samples for 24 h. The 2-NBDG uptake assay was performed with a fluorometer at excitation and emission wavelengths of 485 and 535 nm, respectively. 

### 2.6. Western Blot Analysis

Cells were washed with ice-cold PBS and lysed with lysis buffer (50 mM Tris HCl, 1% Triton X-100, 0.5% sodium deoxycholate, 150 mM NaCl, 1 mM EDTA, 1 mM PMSF, 1 mM sodium orthovanadate, 1 mM NaF, and 0.2% protease inhibitor cocktail; pH 7.2). Protein expression was detected by western blot analysis. 

### 2.7. PPAR-*γ* Transcriptional Activity Assay

HEK293 cells were cotransfected with 1 *μ*g of total DNA-expression plasmids for PPAR-*γ* or retinoid X receptor alpha (RXR-*α*) and luciferase reporter vectors containing PPAR-response elements (PPRE) and *β*-galactosidase. After transfection, the cells were treated with the indicated stimuli for 24 h. PPAR-*γ* and RXR-*α* activities were assessed using the Luciferase Assay System (Promega, Madison, WI, USA). The results were normalized to *β*-galactosidase activity. 

### 2.8. Animal Experiments

Three-week-old male C57BL/6J mice were obtained from Nara Biotech (Seoul, Republic of Korea). Mice were housed in a climate-controlled environment (24 ± 1°C at 50% relative humidity) with 12-h light/12-h dark cycles. The mice were freely fed a 10% fat normal diet (ND, D12450B, Research Diets, New Brunswick, NJ, USA), a 60% kcal high-fat diet (HFD, D12492, Research Diets, New Brunswick, NJ, USA), a 60% kcal high-fat diet plus 1% YPEE, or a 60% kcal high-fat diet plus 5% YPEE. The mice had free access to autoclaved tap water. The diet composition is provided in Table 1. The weight and total food intake of the mice were measured every week. After 9 weeks, the mice were starved for 12 h and blood was drawn from the orbital vein, followed by serum separation. The mice were then sacrificed, and their livers were removed. All animal experiments were performed and approved by the Institutional Animal Care and Use Committee of the Korea Food Research Institute (approval number: KFRI-M-11011, validity date: 18 May 2011).

### 2.9. Insulin Resistance (HOMA-IR)

The homeostasis model assessment of insulin resistance (HOMA-IR) was calculated using the methods described in the paper of Matthews et al., 1985 [15].

### 2.10. Histology Studies

Liver tissue was fixed in 4% buffered formalin and cut into 4 *μ*m thick sections. Sections were stained with hematoxylin and eosin (H&E) and examined by microscopy. 

### 2.11. Quantification of Serum Cholesterol, Triglyceride (TG), Leptin, and Resistin

Cholesterol and triacylglycerol were quantified by enzymatic methods using commercial kits (Asan Pharm, Seoul, Republic of Korea). Leptin and resistin levels were measured using enzyme-linked immunosorbent assay (ELISA) kits (R&D systems, Minneapolis, MN, USA), as per the manufacturer's instructions. 

### 2.12. Statistical Analyses

Statistical analyses were conducted using SPSS 9.0 (SPSS Inc., Chicago, IL, USA). All results are presented as mean ± standard deviation (SD) values. Statistical differences between means were evaluated by one-way analysis of variance (ANOVA) followed by the Bonferroni test. The accepted level of significance was *P* < 0.05 for all analyses in cell and animal experiments. 

## 3. Results

### 3.1. Effect of Ethanol Extract of Yuja Peel (YPEE) on 2-NBDG Uptake in C2C12 Myotubes

**Figure 1:**
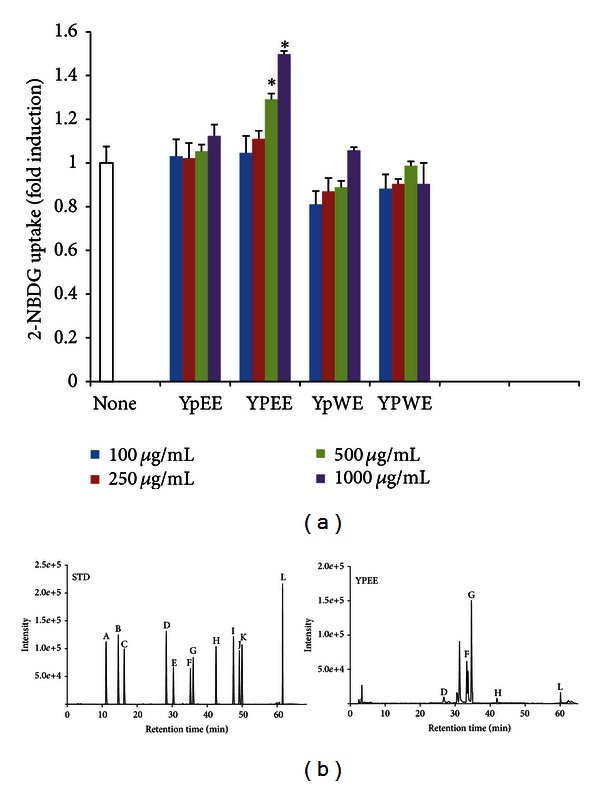
Effect of ethanol extract of yuja pulp (YpEE), ethanol extract of yuja peel (YPEE), water extract of yuja pulp (YpWE), and water extract of yuja peel (YPWE) on glucose uptake in C2C12 myotubes. Cells were treated with the indicated stimulus in a dose-dependent manner, and then, the 2-NBDG uptake assay was performed, as described in Section 2 (a). Data are expressed as mean ± SD. *P < 0.05 versus none. HPLC was performed as described in Section 2. Peaks: A, chlorogenic acid; B, caffeic acid; C, epicatechin; D, rutin; E, luteolin-7-glucoside; F, naringin; G, hesperidin; H, quercetin; I, naringenin; J, kemperol; K, apegenin-7-glucoside; L, Tangeretin. STD, standard; YPEE, ethanol extract of yuja peel (b).

To determine whether yuja extract can stimulate glucose uptake, we first examined its effect on 2-NBDG uptake in C2C12 myotubes. As shown in Figure 1(a), treatment with ethanol extract of yuja peel (YPEE) significantly increased 2-NBDG uptake in a dose-dependent manner. However, ethanol extract of yuja pulp (YpEE) or water extract of yuja peel (YPWE) or pulp (YpWE) did not stimulate glucose uptake. These results suggest that ethanol extract of yuja peel (YPEE) is effective for facilitating glucose uptake. Next we used HPLC to evaluate the flavonoid content of YPEE. As shown in Figure 1(b) and Table 2, the total phenolic content of YPEE was 47.8 ± 0.5 mg/100 g. Among flavonoids contained in YPEE, rutin (2.7 mg/100 g), quercetin (1.7 mg/100 g), tangeretin (0.7 mg/100 g), naringin (11.6 mg/100 g), and hesperidin (36. 3 mg/100 g) appeared to be the major compounds. 

### 3.2. Activation of AMPK and PPAR-*γ* by YPEE

**Figure 2:**
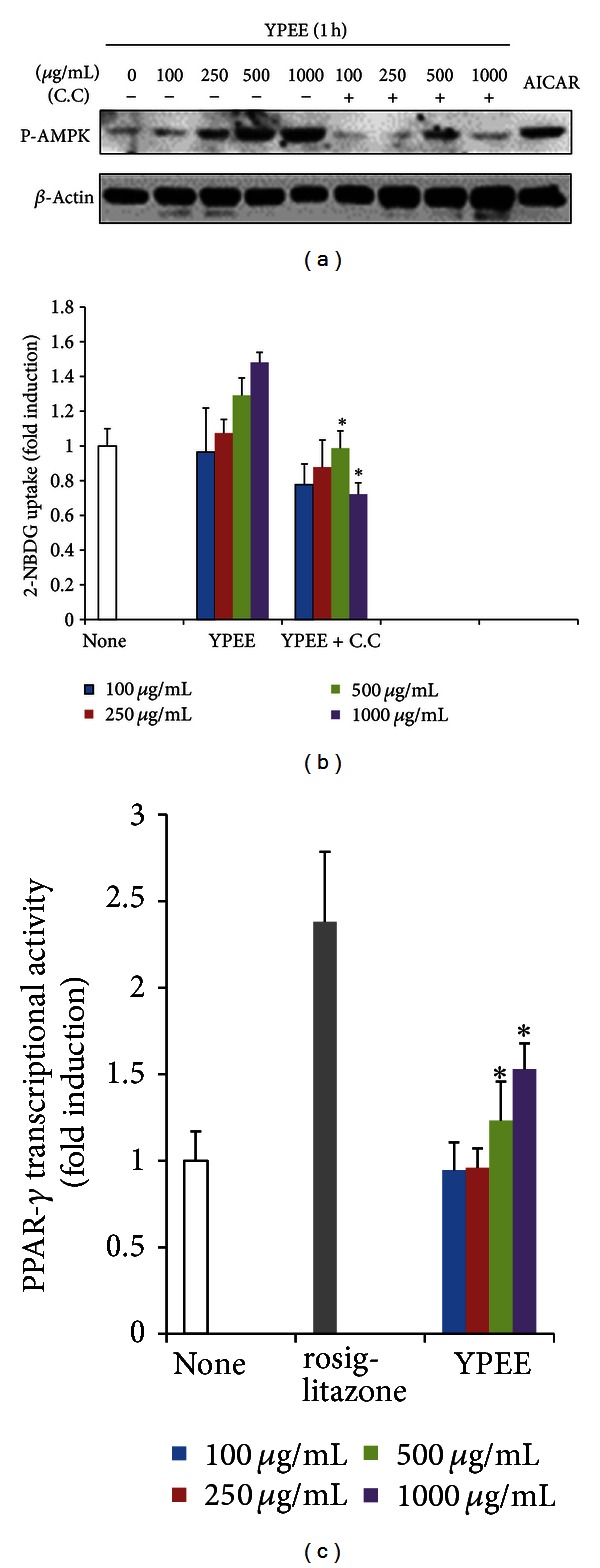
Effect of YPEE on AMPK and PPAR-*γ*. Cells were pretreated with 10 *μ*M C.C for 30 min and consecutively exposed to YPEE for 1 h in a dose-dependent manner. Western blot analysis was performed with phosphospecific AMPK and normal beta-actin antibodies (a). Cells were pretreated with 10 *μ*M C.C for 30 min and consecutively exposed to YPEE, and then, the 2-NBDG uptake assay was performed, as described in Section 2 (b). Data are expressed as mean ± SD. *P < 0.05 versus YPEE. PPAR-*γ* expression vector and PPRE-luc vectors were cotransfected in HEK293 cells and exposed to YPEE or 25 *μ*M rosiglitazone. PPAR-*γ* transcriptional activity was measured with the luciferase assay system (c). Data are expressed as mean ± SD. *P < 0.05 versus none.

Because AMPK and PPAR-*γ* signaling pathways are well-known targets of antidiabetic drugs, we examined whether YPEE-stimulated glucose uptake is accompanied by the activation of AMPK, and PPAR-*γ*. Treatment with YPEE dramatically increased the phosphorylation of AMPK in C2C12 myotubes, and pretreatment with compound C (C.C), a pharmacological inhibitor of AMPK completely decreased the phosphorylations of AMPK stimulated by YPEE (Figure 2(a)). Moreover, pretreatment of compound C inhibited the glucose uptake stimulated by YPEE (Figure 2(b)). 5-amino-1-*β*-Dffff-ribofuranosyl-imidazole-4-carboxamide (AICAR) was used as a positive control of AMPK activation. This result demonstrates that AMPK activation is necessary for the YPEE-stimulated glucose uptake in C2C2 myotubes.

On the other hand, we also examined the transcriptional activity of PPAR-*γ* upon treatment with YPEE. As shown in Figure 2(c), YPEE stimulated the transcriptional activity of PPAR-*γ* in a dose-dependent manner. Combined, these results suggest that YPEE-stimulated glucose uptake is accompanied by the activation of AMPK and PPAR-*γ*.

### 3.3. Effect of YPEE Supplementation on Body Weight, Liver Fat Contents, Serum TG, and Total Cholesterol Levels

**Figure 3:**
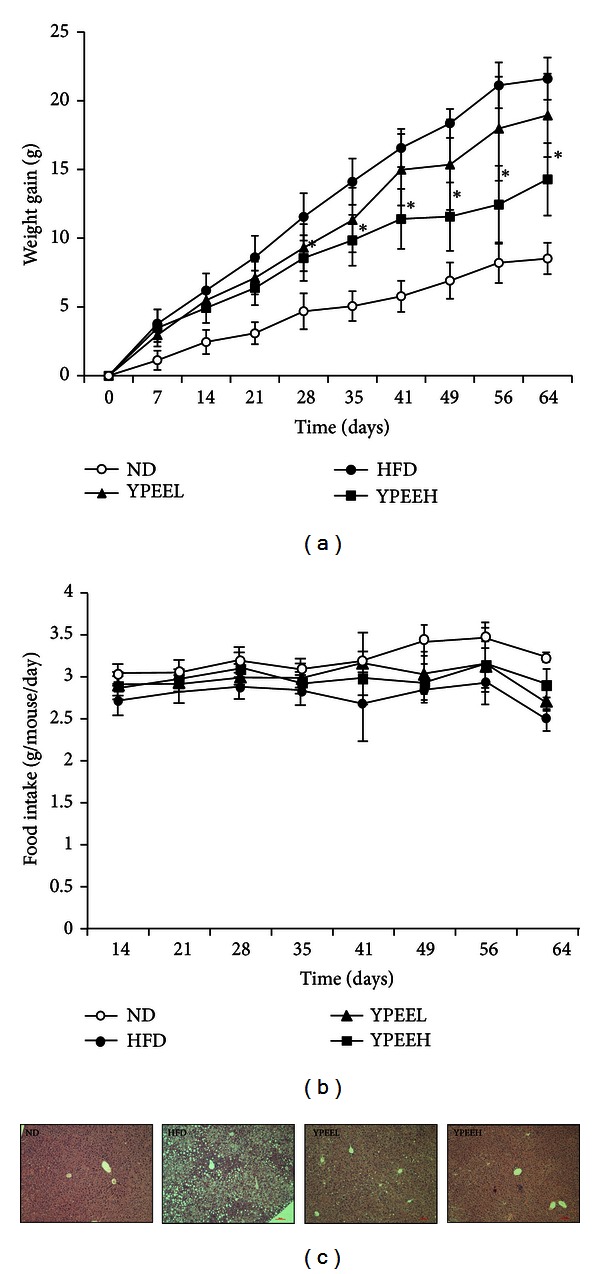
Effect of YPEE on body weight gain, food intake, and liver fat accumulation. Mice were fed a normal diet (ND), high-fat diet (HFD), high-fat diet + 1% YPEE (YPEEL), or high-fat diet + 5% YPEE (YPEEH). Body weight gain (a), food intake (b), and liver fat accumulation (c) were measured, as described in Section 2. Data are expressed as mean ± SD.*P < 0.05 versus HFD.

To further assess the *in vitro *antidiabetic effects of YPEE, we employed a HFD-induced obese mouse model. The initial body weights of four groups were not statistically different; however, final body weights were significantly lower in the YPEEH group (Table 3). The total food-intake values showed no significant changes (Figure 3(b)) in all the groups. As shown in Figure 3(a), the body weight gain in the HFD group was higher than that in the normal diet (ND) group. Under identical conditions, higher intake of YPEE (5%) induced greater weight loss. Levels of cholesterol and TG in serum were significantly higher in the HFD group than in the ND group, yet this was alleviated significantly by YPEE supplementation (Table 3). Serum glutamic oxaloacetic transaminase (GOT) and glutamic pyruvic transaminase (GPT) levels were not significantly changed in the YPEE group compared with the ND group (data not shown). In addition, liver tissue fat was significantly decreased in the HFD plus YPEE group compared with the HFD group (Figure 3(c)). 

### 3.4. Effect of YPEE on HOMA-IR and Secretion of Adipocytokines

The YPEE group showed lower serum glucose and insulin levels than the HFD group (Table 3). On the basis of these results, HOMA-IR was analyzed to evaluate insulin resistance level in this study. As shown in Table 3, insulin resistance (HOMA-IR) was significantly increased in the HFD group compared with the ND group. Administration of 5% YPEE resulted in a decrease in insulin resistance compared with the HFD group.

**Figure 4:**
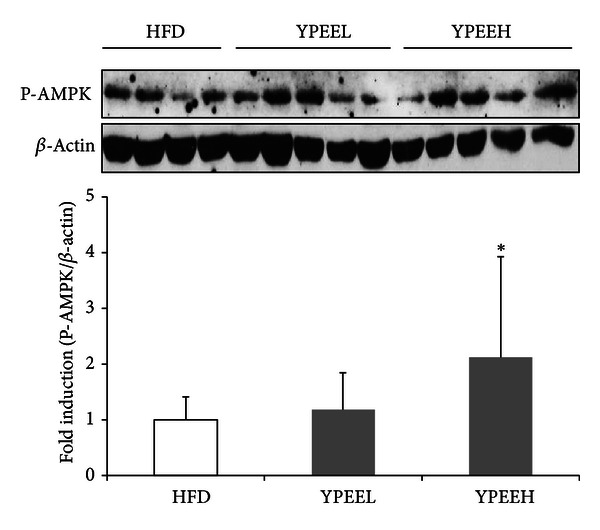
Phosphorylation of AMPK by YPEE in mouse muscle tissue. After finishing animal experiments, the muscle tissues randomly collected and were homogenized with lysis buffer. AMPK phosphorylation was measured using western blot analysis, and bands were analyzed by densitometry. Data are expressed as mean ± SD. *P < 0.05 versus HFD.

Because it is well known that obesity-induced adipocytokine release is important event in insulin resistance, we next examined the effect of YPEE on adipocytokine production increased by HFD. As shown in Table 3, levels of adipocytokines, including leptin and resistin, were increased in the HFD group relative to the ND group. Addition of 5% YPEE to the HFD resulted in significantly decreased levels of leptin. To determine whether AMPK was phosphorylated in the muscle tissue of the mice fed HFD plus 5% YPEE, we performed a Western blot analysis using randomly collected muscle tissue. HFD plus 5% YPEE resulted in increased phosphorylation of AMPK compared with HFD alone (Figure 4). These results show that the antidiabetic effect of YPEE appears to be mediated by AMPK in these mouse models, in addition to the cell culture system.

## 4. Discussion 

The aim of this study was to evaluate the antidiabetic effect of yuja in a cell culture system and in HFD-fed mice. We found that YPEE treatment produced antidiabetic effects in both C2C12 myotubes and mouse models. In contrast, YpEE, YPWE, and YpWE did not have antidiabetic effects. In addition, AMPK and PPAR-*γ* were significantly activated by YPEE in our studies, suggesting that YPEE has an antidiabetic effect via activation of the AMPK and PPAR-*γ* signaling pathways.

Natural plants generally contain high polyphenol content, which has shown positive effects for the prevention of metabolic disorders such as diabetes [16, 17]. Thus, the antidiabetic effect of yuja peels is likely to be related to its phenolic composition, which comprises several major compounds, including rutin, quercetin, tangeretin, naringin, and hesperidin, as shown in Table 2 and Figure 1(b). Rutin has been reported to exert beneficial health effects in a variety of disease models, such as inflammation, cancer, oxidative stress, and cardiovascular diseases [18–21]. Meanwhile, quercetin, naringin, and hesperidin have also been reported to exhibit beneficial health effects [22–24]. Quercetin has been suggested to prevent the development of various diseases, such as viral infection, cancer, inflammation, and metabolic syndrome [22, 25]. Hesperidin and tangeretin, which are found in citrus fruits such as yuja, also exhibit beneficial health effects against certain diseases, such as hypercholesterolemia, obesity, and diabetes [24–26]. Our previous study also has shown the ability of tangeretin to reduce the levels of circulating lipid mediators, including TG and total cholesterol, in obese mice [26]. Taken together, these results suggest that the phenolic compounds of YPEE can improve insulin resistance and thereby contribute to the amelioration of dyslipidemia, a metabolic complication of diabetes.

To the best of our knowledge, the antidiabetic effect of YPEE arises from the presence of certain flavonoids. However, we did not identify the precise phenolic compounds involved in the antidiabetic effects exerted by YPEE. Further investigations are needed to illustrate the relationship between the major compounds and the antidiabetic effect stimulated by YPEE.

Moreover, it has been reported that yuja peel contains abundant fiber, which can reduce blood glucose and improve insulin resistance [27, 28]. Yuja also contains carotenoids, which have been proposed to improve insulin resistance by stimulating insulin-signaling pathways [29]. In the present study, we speculated that like flavonoids, the minor compounds, such as fiber, carotenoids, and ascorbic acid, present in YPEE contribute to the antidiabetic effect of yuja, but we did not elucidate the underlying mechanism. AMPK and PPAR-*γ* were significantly activated by YPEE in myotubes. AMPK plays a central role in the metabolic process [4, 5]. Thus, many investigators have focused on the role of AMPK in the development of antidiabetic compounds. In addition, several natural ingredients have been shown to exert antidiabetic effects, accompanied by AMPK activation [4, 5]. The extract of *Malva verticillata* (MV) seeds improves diabetes by increasing glucose uptake via AMPK, dependent signaling pathways [30]. Another study demonstrated that chitosan, a natural biomaterial, exerts a glucose-lowering effect by activating AMPK and Akt in streptozotocin-induced diabetic rats [31]. The authors suggested that the mechanism of the antidiabetic effect is similar to that of metformin, an antidiabetic drug that activates AMPK. This is in consensus with our results concerning the antidiabetic activity of YPEE that activated the AMPK signaling pathway (Figure 2(a)). YPEE also increased the transcriptional activity of PPAR-*γ* (Figure 2(c)), another well-known antidiabetic target. Numerous studies have demonstrated that targeting PPAR-*γ* is one of the best strategies for developing antidiabetic drugs [6, 32]. In this study, YPEE exerted an antidiabetic effect by activating PPAR-*γ*. However, the precise upstream and downstream regulators of AMPK and PPAR-*γ* stimulated by YPEE remain unclear. Further studies should be performed to clearly determine how YPEE regulates AMPK and PPAR-*γ*.

A 5% YPEE reduced fat accumulation and adipocytokine production and improved insulin resistance (Figure 3 and Table 3). A number of studies have suggested that adipocyte-derived cytokines are important factors in the etiology of diabetes. In diabetic patients, the levels of several cytokines, including resistin and leptin, are significantly increased [33, 34]. As shown in Table 3, the YPEEH appears to partially and significantly counteract the negative metabolic effects of the HFD corresponding with previous studies. Moreover, YPEE also increased the phosphorylation of AMPK in mouse muscle tissue, similar to cell culture systems. Thus, we speculate that YPEE might alter inflammatory factors or inflammation-induced insulin resistance, and that AMPK might play an important role in this action. Further studies are needed to determine the correlation between adipocytokine secretion and AMPK activity upon treatment with YPEE.

## 5. Conclusion

This study is the first to demonstrate the antidiabetic activity of YPEE by targeting AMPK and PPAR-*γ* signaling in myotubes. Moreover, YPEE also improved the abnormal levels of blood glucose, lipid, and adipocytokines in the HFD-fed mice. In addition, the major phenolic compounds in YPEE were found to include rutin, quercetin, naringin, tangeretin, and hesperidin compounds with known health promoting effects or antidiabetic effects. Therefore, YPEE may be useful for preventing diabetes and related diseases. 

## Figures and Tables

**Table 1:** Composition of the diets.

Ingredients	ND (g)	HFD (g)	YPEEL (g)	YPEEH (g)
YPEE	0	0	25	125
Casein, lactic	473.9	646.1	646.1	646.1
L-Cystine	7.1	9.7	9.7	9.7
Corn starch	746.4	0.0	0.0	0.0
Maltodextrin 10	82.9	403.8	403.8	403.8
Sucrose	829.3	222.3	222.3	222.3
Cellulose, BW200	118.5	161.5	161.5	161.5
Soybean oil	59.2	80.8	80.8	80.8
Lard	47.4	791.5	791.5	791.5
ETC	135.2	184.3	184.3	184.3

Total	2500	2500	2525	2625

ND: normal diet; HFD: high-fat diet; YPEEL: high-fat diet plus 1% ethanol extract of yuja peel/kg diet; YPEEH: high-fat diet plus 5% ethanol extract of yuja peel/kg diet. ETC: et cetera.

**Table 2:** Total phenolic compounds and major compounds content, expressed as mg/100 g of YPEE.

	Total phenolic compounds	Rutin	Naringin	Hesperidin	Quercetin	Tangeretin
YPEE	47.8 ± 0.5	2.7 ± 0.0	11.6 ± 1.7	36.3 ± 4.3	1.7 ± 0.1	0.7 ± 0.0

All experiments were performed with 3 replicates. Data are expressed as mean ± SD.

**Table 3:** Inhibitory effect of YPEE on high-fat diet-induced cholesterol, TG, resistin, leptin, glucose, and insulin resistance (HOMA-IR).

	ND	HFD	YPEEL	YPEEH
Initial body weight (g)	20.2 ± 1.1	19.9 ± 0.8	20.1 ± 0.9	18.9 ± 1.2
Final body weight (g)	27.8 ± 1.6	40.4 ± 2.0^a^	38.4 ± 3.8	32.4 ± 2.4^b^
HOMA-IR	1.95 ± 0.5	26.2 ± 5.4^a^	24.2 ± 6.4^b^	7.91 ± 3.3^b^
Glucose (mg/dL)	115 ± 3	187 ± 9^a^	195 ± 8^b^	174 ± 11^b^
Serum				
Resistin (ng/mL)	17.5 ± 1.9	29.9 ± 2.1^a^	29.8 ± 3.3	20.6 ± 1.8^b^
Leptin (ng/mL)	1.6 ± 0.2	14.0 ± 1.6^a^	10.1 ± 1.5^b^	3.7 ± 0.9^b^
Total cholesterol (mg/dL)	79.1 ± 3.0	92.0 ± 3.1^a^	77.6 ± 1.7^b^	74.2 ± 1.2^b^
Triglyceride (mg/dL)	68.4 ± 9.8	78.5 ± 6.4^a^	75.6 ± 4.8	67.5 ± 8.4^b^
Insulin (ng/mL)	0.23 ± 0.05	1.85 ± 0.33^a^	1.64 ± 0.39^b^	0.58 ± 0.21^b^

ND: normal diet; HFD: high-fat diet; YPEEL: high-fat diet plus 1% ethanol extract of yuja peel/kg diet; YPEEH, high-fat diet plus 5% ethanol extract of yuja peel/kg diet. Data are expressed as mean ± SD (*n* = 8).^ a^ *P* < 0.05 versus ND; ^b^ *P* < 0.05 versus HFD.
